# Referrals to a pediatric emergency department of a tertiary care teaching hospital before and after introduction of a referral education module - a quality improvement study

**DOI:** 10.1186/s12913-020-05649-w

**Published:** 2020-08-17

**Authors:** Gopalakrishnan Ezhumalai, Muralidharan Jayashree, Karthi Nallasamy, Arun Bansal, Bhavneet Bharti

**Affiliations:** Department of Pediatrics, Advanced Pediatrics Centre, Postgraduate Institute of Medical Education and Research (PGIMER), Chandigarh, India

**Keywords:** Referral letter, Quality of referral, Pediatric emergency, Triage

## Abstract

**Background:**

Provision of timely care to critically ill children is essential for good outcome. Referral from smaller peripheral hospitals to higher centers for intensive care is common. However, lack of an organized referral and feedback system compromises optimal care. We studied the quality of referral letters coming to our Emergency Department (ED) with respect to their demography, association with severity of illness and mortality before and after referral education.

**Methods:**

Our study was completed in three phases in the Pediatric ED; Pre-intervention, Intervention and Post intervention phases. Quality of referral letter was matched with a quality checklist proforma and graded as ‘good’, ‘fair’ and ‘poor’ if it scored > 7, 5–7 and < 5 points respectively. A peer reviewed referral education module was prepared using case studies, expert opinions, and lacunae observed in the first phase and administered to health care providers (HCP’s) of referring hospitals. Quality of referral letter was compared between pre and post intervention phases.

**Results:**

Most referrals belonged to the neighboring states of Punjab (48.2%) and Haryana (22.4%). Major referring hospitals were from public sector (80.9%), of which the teaching hospitals topped the list (53.6%). Government run ambulance services (85.5%) was commonest mode of transport used and need for a PICU bed and/or mechanical ventilation (50.4%) was the commonest reason for referral. The post intervention phase saw a significant decline in the proportion of poor (93.2 vs.78.2%; *p* = 0.001) and a significant increase in the proportion of fair (6.1 vs 18%; *p* = 0.001) and good referral letters (0.7 vs 18%; p = 0.001). The proportion of children with physiological decompensation at triage had reduced significantly in the post intervention phase [513 out of 1403 (36.5%) vs. 310 out of 957 (32.3%); p = 0.001].

**Conclusion:**

Referral education had significantly improved the quality of referral letters. Proportion of children with physiological decompensation at triage had decreased significantly after referral module. This change suggests sensitization of the peripheral hospitals towards a better referral process. Continued multifaceted approach will be required for sustained and increased benefits.

## Background

Provision of timely and optimal care to critically ill children is crucial for good outcome. With development of specialized pediatric critical care units, referral from community, district or other peripheral healthcare facilities has gained greater momentum [1]. Referral is a process in continuum; beginning from the referring and ending at the referred facility [2]. Maintenance of optimal physiology during referral is vital [3, 4]. India unfortunately lacks an organized referral network. This is further compounded by multiple barriers which include poor referral process and transport facilities [5]. A study by Praveen K et al. from a tertiary care referral hospital on brought in dead cases, found that lack of prereferral communication, poor documentation and inadequate transport services to address resuscitation needs were the common factors identified in children with decompensated referrals [6]. In another study from India by Sankar et al., auto rickshaw was the most common mode of transport used for referral and none of the children referred had a proper documentation or pre-referral communication [7].

Our hospital is a 300 bedded pediatric multispecialty teaching and referral center which caters to patients from 5 neighboring northern states viz. Punjab, Haryana, Himachal Pradesh, Rajasthan and Uttar Pradesh. The standalone Emergency Department (ED) of the hospital is one of the busiest among the pediatric services. It records about 25,000 patient visits per year, of which about 10,000 get admitted. Most of these are patients referred from public (community health centers, district hospitals, and medical colleges) and corporate sector hospitals of the adjoining states. Since there is no standard referral system in India, a significant proportion of these referred cases are critically ill at arrival. Lack of pre-referral communication, overwhelms the referred facility (infrastructure and personnel), creating mismatch between demand and supply. Decompensated referrals and inadequate documentation remain constant challenges in providing acute care. As a first step to streamline our emergency referrals, we conducted this study to ascertain the quality of referral letters accompanying patients coming to our ED and their association with severity of illness and mortality before and after introduction of a referral education module. Using this data, we plan to establish a standard referral and feedback system to optimize care.

## Methods

We conducted a prospective before and after study which was divided into three phases; Pre- intervention (Phase-I), Intervention (Phase II) and Post intervention (Phase III). All consecutive children aged > 1 month to 12 years of age referred to our ED during the study period were included.

Our ED uses a “five level” triage in which patients are assigned levels, based on their disease acuity and physiological status [8]. The triaging is done by a mid-level MD Pediatrics trainee who is a certified Advanced Life Support (ALS) provider, supported by a post-doctoral senior resident who is also ALS certified. The management priorities are based on the triage level assigned and urgency of care. Children with lesser acuity of illness are either counselled to wait for further diagnostic evaluations; or sent home after adequate treatment with advice to attend the out-patient department. Those who get admitted are stabilized and managed in the emergency ward till they are transferred out to the respective inpatient wards.

The pre- intervention phase lasted for 4 months from January 2016 to April 2016. During this phase a total 1403 children referred from different hospitals were studied. The referral letters accompanying these children were collected and studied for quality and completeness by comparing them with a quality checklist performa. The comparator quality checklist performa was made a priori based on WHO recommendations [9]. The quality of each referral letter was estimated by granting a score of 1 to the presence of each item in the performa. There were a total of 10 items which included i) Details of the referring center ii) Reason for referral iii) General information of patient iv) Clinical examination findings v) Details of investigations performed vi) Provisional diagnosis vii) Documentation of patient condition prior to referral viii) Documentation of stabilization and treatment received prior to referral ix) Details of treatment during transport if any and x) Communication prior to referral with both referred facility and information on caregiver counselling [9]. Some items were subdivided into their integral components and each component was granted equal scores between 0.2 and 0.5 in accordance with the number of components. All the components included were given equal importance. Each referral letter was given an objective score and graded as ‘good’ if it scored > 7 points, ‘fair’ if it scored between 5 and 7 and ‘poor’ if the score was less than 5. The maximum score a referral letter could get was 10.

During Phase-II **(**May to August 2016) **w**e developed the referral education module with inputs from emergency and social pediatrics experts (MJ, AB, BB, KN). The, lacunae in referrals and referral letters observed during the first phase were analyzed and discussed. Components to address these problems were also included in the module content. Anonymized case studies (identity of patient, referring hospital and physician were kept confidential), were prepared to highlight both good referral and common errors during referrals. The content of the module emphasized on 1) How to identify a sick child who needs referral 2) importance of timely referral 3) key components to be addressed during the referral process like stabilization and maintenance of airway, breathing and circulation, fluids and medications 4) Importance of good pre-referral communication 5) Importance of a good written documentation (referral letter) and feedback. This module was reviewed by all the co-investigators before finalizing it. The final module (duration 2 h) was then administered onsite to all the doctors, and nurses working in the ED of various hospitals from where the maximum referrals had taken place in Phase I. The list of hospitals to be visited was prepared and the concerned hospitals were informed at least 1 week prior to the scheduled visit to ensure maximum participation from concerned health care providers of that site. On the proposed date and time, the team of investigators visited the site and administered the module to the target group. Written handouts emphasizing key aspects of a good referral, were also given. In the post intervention phase, which lasted for 5 months from September 2016 to January 2017, a total 957 referred cases were studied. The referral letters and their quality were assessed once again with the same quality checklist performa and objectively scored similar to that described for Phase I.

The pre and post intervention phases were then compared with respect to demography of referral letters, association with severity of illness and mortality using Chi-square test or Student’s t test as applicable. Quality of referral letter is expressed as proportion. *P* <  0.05 was taken as significant.

### Outcome parameters

Change in proportion of good, fair and poor-quality referral letters before and after the referral education module was taken as primary outcome. Reasons for referral, proportion of patients transported in ambulance with a trained personnel, association between the quality of referral and triage physiological status were secondary outcome parameters.

## Results

A total of 3231 admissions, 1902 during pre-intervention and 1329 during post intervention were screened. Of them, 1403 and 957 referred cases were included in the pre and post intervention phases respectively. The demographic data, type of referring facility, indications for referral and mode of transport are given in Table 1. The median age and proportion of boys were similar in both phases. About two thirds of referrals (67.4%) were from rural background.

**Table 1:** Comparison of demography, details of referring facility, indications and transport in pre vs post intervention phases

	Preintervention *N* = 1403	Post intervention *N* = 957	*P* value
**Age** Median (IQR)	3.6 (1.9–7)	3.8 (2.3–7)	0.04
**Boys** N (%)	860 (61.3)	574 (60)	0.52
**Residential area**			
**Rural** N (%)	969 (69)	623 (65)	0.04
**Urban** N (%)	434 (31)	334 (35)	
**Referring States**			
Punjab	695 (49.5)	450 (47)	0.23
Haryana	313 (22.3)	194 (20.3)	0.24
Chandigarh	190 (13.6)	154 (16.1)	0.08
Himachal Pradesh	147 (10.5)	91 (9.5)	0.44
Uttar Pradesh	58 (4.1)	68 (7.1)	0.002
**Type of referring facility**			
***Public sector***	1198 (85.4%)	799 (83.5%)	0.21
Medical College & Teaching Hospital	664(47.3%)	397(41.4%)	0.005
Community Health Care Centers	317(22.5%)	205(21.4%)	0.50
District Hospitals	217(15.4%)	197(20.5%)	0.001
***Corporate sector***	205 (14.6%)	158 (16.5%)	0.21
Private Hospitals	186(13.2%)	147(15.3%)	0.15
Medical College & Teaching Hospitals	19(0.01%)	11(0.01%)	0.66
**Mode of transport**			
Government ambulance services	1200 (85.5)	820 (85.6)	0.91
Private ambulance services	147 (10.5)	95 (10)	0.66
Self-owned vehicles	56 (4)	42 (4.4)	0.63
**Reasons for referral**			
Need for PICU /Mechanical ventilation	654 (46.6)	482 (50.4)	0.07
Expert consultation	569 (40.5)	343 (35.8)	0.02
Financial constraints	140 (10)	101 (10.6)	0.65
Parental request	40 (2.9)	31 (3.2)	0.47

Foot note: Rural, Urban description adapted from Indian census 2011 [10]

Most patients were referred from public sector hospitals. Government run ambulance services was the most common mode of transport availed in both phases [pre-intervention 1200 (85.5%) vs post intervention 820 (85.7%)], followed by private ambulance services [pre intervention 147(10.5%) vs post intervention 95(9.9%)] and self-owned four wheeler [pre-intervention 56 (4%) vs post intervention 42 (4.4%)]. However, only 7 and 8 referrals in both phases respectively had a healthcare provider (HCP) accompanying the patients till the referred facility. Half of the patient referrals were for intensive care [654 (46.6%) vs 482 (50.4%)] of which [185 (28%) vs 195 (40%)] were for mechanical ventilation. The other indications were: need for expert diagnostic opinion, financial constraints and parental request (Table 1).

### Quality of referral letters

Majority of the referral letters in the pre-intervention phase were poor (*n* = 1308, 93.2%); only 10 (0.7%) were graded as good. Most referral letters had inadequate information related to pre-referral clinical examination findings, investigations and diagnosis. Only a very small proportion (3.7%; *n* = 52) had appropriate pre-referral documentation of stabilization measures and treatment. There was lack of pre-referral communication with the referred facility, as well as with parents/ guardians regarding the need of referral and risk of transport. This information was not documented in the referral letters.

In the post intervention phase, the proportion of poor referral letters had significantly decreased (pre-intervention 93.2% vs. post intervention 78.2%; *p* = 0.001) and the good and fair referral letters had significantly increased [pre-intervention 0.7% vs. post intervention 4%; p = 0.001 and 6.1% Vs 17.8%; p = 0.001respectively]. Pre-referral documentation in different domains although still inadequate had significantly improved in the post intervention phase; documentation of clinical findings, diagnosis and investigations (0.2% vs 19.5%, *P* <  0.001) and information related to stabilization and treatment had improved significantly in the post intervention phase (3.7 vs. 23.8%; *p* = 0.0001). Although caregiver counselling had improved, details regarding communication with referred facility remained unchanged in the post intervention phase. The comparative data on quality of referral letters in both the phases is as depicted in the Table 2.

**Table 2:** Comparison of quality of referral letters

Components of referral	Pre intervention phase n (%)	Post intervention phase n (%)	*P* value
Documentation of clinical findings and/or working diagnosis	3 (0.2)	187 (19.5)	< 0.001
Documentation of pre-referral investigations	3 (0.2)	184 (19.2)	< 0.001
Documentation of pre-referral stabilization / treatment	52 (3.7)	228 (23.8)	< 0.001
Pre-referral communication with referred facility	0	0	
Information on caregiver counselling	0	10 (1)	< 0.001
Transport details	0	16 (1.7)	< 0.001
Healthcare provider accompanying transport	7 (0.5)	8 (0.8)	0.44
Overall referral letter score			
Median (IQR)	3.75 (2.75–4.0)	4.0 (3.0–4.75)	< 0.001
Good (8–10)	10 (0.7)	38 (4)	
Fair (5–7)	86 (6.1)	171 (17.8)	< 0.001
Poor (0–4)	1307 (93.2)	748 (78.2)	

We had visited 9 out of 36 health care facilities that had referred to us during the pre-intervention phase for administering the intervention module. This was based on the maximum number of referrals we had received from these hospitals (912 out of 1403; 65%). In the post -intervention phase 704 out of 957 referrals (73.5%) were from hospitals that had received the intervention. The analysis of this subgroup also showed a consistent and significant improvement in quality of referral letters in the post intervention phase. The proportion of poor referral letters had decreased significantly from 835 out of 912 (91.6%) to 510 out of 704 (72.4%); *p* = 0.001.

Severity of illness as classified according to physiological status at the time of triage was similar in both phases (Table 3). Proportion of children arriving in a physiologically decompensated state i.e. respiratory failure, hypotensive shock, and cardiopulmonary failure combined had reduced significantly slightly in the post intervention phase [513 out of 1403 (36.5%) vs. 310 out of 957 (32.3%); p = 0.001) On the other hand, the proportion of children brought in cardiopulmonary arrest was similar in both phases [Pre-intervention 56 (4%) vs post intervention 48 (5%); *p* = 0.45]. There was a significant drop in decompensated referrals among those with poor quality referrals in the post intervention phase [471 out of 1308 (36%) vs. 233 out of 748 (31%); *p* = 0.02]. In-hospital mortality also did not differ between both phases [Pre-intervention 107 (7.6%) vs post intervention 70 (7.3%); *p* = 0.11].

**Table 3:** Overall Physiological Status and clinical outcome in Pre vs Post intervention phases

Status	Pre-intervention n(%)	Post-intervention n (%)	***P*** value
Compensated Referrals	834 (59.4)	600 (62.7)	0.11
Respiratory distress	193 (15.1)	157 (16.4)	
Compensated shock	100 (7.2)	94 (9.8)	
Primary brain dysfunction	541 (38.5)	349 (36.5)	
Decompensated Referrals	513 (36.5)	309 (32.3)	0.03
Respiratory failure	283 (20.5)	174 (18.2)	
Hypotensive shock	215 (13.7)	130 (13.6)	
Cardiopulmonary failure	15 (1)	5 (0.5)	
Cardiopulmonary arrest	56 (4)	48 (5)	0.23
ER mortality	107 (7.6)	70 (7.3)	0.11

## Discussion

This study was designed firstly to observe the quality of referral letters accompanying children who are referred to our pediatric ED and secondly to develop a referral educa tion module for health care providers at different referring healthcare facilities; and thirdly to assess the impact of this intervention on the quality of referral letters.

A significant proportion (73%) of those who required admission were ‘referred’ patients, primarily from public sector teaching hospitals. A study from another teaching and tertiary care hospital has reported findings to the contrary; only 22.5% of the referred patients required admission [7]. The higher admission rate among referred patients in our set up is related to multiple factors. Ours is the only tertiary level subspecialty public sector hospital catering to 5 neighboring states. Lack of organized emergency and intensive care services and expertise in the peripheral hospitals, causes our hospital to bear the brunt of large number of referrals and admissions.

Referral is a process in continuum; care of the patient during transport is key to good outcome. This requires fully equipped ambulance manned by HCP trained in basic and advanced life support skills. Although Government run ambulances were availed commonly in our study, the critically ill patient was unaccompanied by HCP in most situations. The fact that 4.4% of referral admissions were received in cardiopulmonary arrest draws attention to this fact that there was a complete lack of resuscitation and stabilization enroute. A recent study from our centre, showed that about 3% emergency department admissions were brought in dead. Clinical deterioration was noted in 62% children during transport, only 5 received CPR enroute [6]. Another study by Bhalla et al. from Delhi which looked into the care given during transport of trauma patients, found several medicolegal issues and barriers for care during referral [11].

Accurate transfer of patient information in the form of a well-documented referral letter is crucial for maintaining continuity of care especially in sick children. Lack of vital information with respect to clinical status and treatment received, poses lot of problems for the referred facility. Since most patients in our setting lack knowledge or information about the treatment given, the healthcare providers at the receiving facility are highly dependent on the referring doctor’s documentation or verbal information. We found that almost all referral letters lacked the most essential information required for maintaining continuity of care [7]. The referral letters in the pre-intervention phase had missing information related to illness, clinical examination, treatment given, investigations, procedures and pre-referral stabilization. Studies have shown that nearly one third of specialist referrals from general practitioner (GP), lack essential clinical information and are often inadequate [12–14]. Referral note without adequate information was found in 69% of brought in dead referrals at a tertiary pediatric ED in India [6]. Another study evaluating the quality of GP referrals to a South African tertiary care hospital, reported that certain important components related to pre referral treatment (6.3%), laboratory tests (8.3%) and special tests (4%) were mentioned in very few referral letters only [15].

The reasons for referral were documented in most referral letters in both phases. Our findings compare favorably, with that reported by Langalibaele et al. [15] and Lachman et al. [16] which was 88 and 100% respectively. But pre-referral communication with the referred center was absent. In a resource limited setting like ours, absence of prior information results in poor preparedness at the tertiary level, the case in example being need of a ventilator.

We observed that all the referral letters were hand written and unstructured. Sankar et al. found that almost two third of their referral letters were incomplete and lacked crucial information [7]. Majority of the referral letters were in the ‘letter format’ similar to that reported by Xiang et al. [14]. Lack of structured format could have been one of the reasons for the deficiency of several domains in these referral letters similar to that reported by Manis et al. [17, 18]. A structured format is preferable as it ensures completeness of information by forcing the health care provider to fill all the columns and check all the required boxes.

Various methods have been tried worldwide to improve the incorporation of relevant information in referral letters. Referral guidelines, structured performa, peer or specialist feedback, risk factor checklist, and referral management scheme (RMS), have been some of the measures tried to improve referral content [14]. In the index study we introduced a referral education module which did make a significant impact. The proportion of poor referral letters had significantly reduced along with a corresponding significant increase in proportion of fair and good referral letters. Referral content with respect to clinical examination findings, and pre-referral stabilization and treatment during transport had improved significantly. The proportion of patients received in a physiologically decompensated state had significantly decreased in the post intervention period. Furthermore, the proportion of referrals from teaching hospitals had decreased significantly in the post intervention phase. Educational interventions thus improved the quality of referral letters, decreased overall referrals and decompensated referrals. Although we cannot definitively conclude on the causality, our findings suggest that our module has sensitized the healthcare providers in peripheral healthcare facilities and teaching hospitals about the need for timely stabilization, and good quality referral letter. It has initiated a dialogue between the referring centers and our facility and has strengthened the back-referral process i.e. sending back stabilized patients to the primary referring facility.

The strength of our study is that it is first of its kind in a setting which lacks an organized referral network. We included all consecutive referrals to achieve a sizeable sample in both phases. The information obtained in our study paves the way for development of an organized referral network which will connect tertiary facilities with the other public and corporate hospitals in the periphery.

The major limitation of our study was that we could not cover all the health care facilities that were referring to us as they were distributed over a wide geographical area. We had targeted our interventions on hospitals from where the maximum referrals were received during the pre-intervention phase. We feel that the intervention phase was short, considering that educational interventions may have to be sustained to achieve greater impact in changing practice. Hence, the initial sensitization achieved by our study should be further consolidated by a more sustained multifaceted outreach and continuous feedback process at a policy level.

Lastly, being a before and after study design with inherent limitation of Hawthorne effect, the estimate of effect in the post intervention phase cannot be completely attributed to interventional measures. The reduction in number of referrals in the post intervention phase could be attributed to a natural frequency of admission during this phase or a Hawthorne effect.

## Conclusion

Referral education of healthcare providers had improved the quality of referral letters by improving some domains of referral content. Proportion of children presenting to triage with physiological decompensation had significantly decreased after introduction of educational interventions. This change suggests sensitization of the peripheral hospitals towards a better referral process. Continued multifaceted approach will be required for sustained and increased benefits. This will pave the way for building an organized referral and back referral network among the healthcare facilities in this particular geographic region and thus reduce overburdening of tertiary care facilities.

## Data Availability

The datasets generated and/or analysed during the current study are not publicly available because the data contains names of the referring healthcare facilities, but are available from the corresponding author on reasonable request.

## Abbreviations

ED: Emergency Department
WHO: World Health Organization
GP: General Practitioner
HCP: Health Care Providers
MD: Doctor of Medicine
ALS: Advanced Life Support
IMCI: Integrated Management of Childhood Illness
RMC: Referral Management Centres
PICU: Pediatric Intensive Care Unit
IQR: Interquartile Range
PGIMER: Post Graduate Institute of Medical Education and Research
